# Engineered bone matrix models for understanding breast cancer skeletal metastasis

**DOI:** 10.1007/s10555-025-10310-1

**Published:** 2026-01-24

**Authors:** Kylie M. Persson, Matthew A. Whitman, Claudia Fischbach

**Affiliations:** 1 Meinig School of Biomedical Engineering, Cornell University, Ithaca, NY USA; 2 Kavli Institute at Cornell for Nanoscale Science, Cornell University, Ithaca, NY USA

**Keywords:** Bone extracellular matrix, Bone metastasis, Breast cancer, Tissue engineering, Mineralization, Collagen

## Abstract

Bone is the most common site of metastasis in patients with advanced breast cancer and serves as a reservoir from which secondary metastases often originate. While research has traditionally focused on understanding the biochemical signals involved in bone metastasis, bone matrix changes are inextricably linked with its pathogenesis as well. Indeed, decreased bone matrix density is both a symptom of late-stage disease and a risk factor for metastasis formation. Vice versa, raising bone density protects against metastasis. How bone matrix controls metastatic outgrowth and progression and which biochemical or biophysical mechanisms are involved in these processes is poorly understood due in part to limitations in current models of bone metastasis. In this review, we discuss the role of bone matrix in the microenvironmental regulation of bone metastasis and its effect on mechanosignaling and highlight current and future engineered model systems that have the potential to yield new mechanistic insights to advance the clinical prognosis of breast cancer patients.

## Introduction

Breast cancer represents about 30% of new cancer diagnoses for women in the USA and was the most common newly diagnosed cancer in 2024 [1]. Although 5-year survival rates for patients with primary breast cancer have risen to an impressive 90%, they drop to just 30% for patients with distant metastases [2]. The skeleton is the most common site for breast cancer metastasis and affects approximately 80% of patients with high grade disease. Although treatments exist to restrict the progression of metastatic lesions and palliate symptoms, bone metastases are incurable and associated with poor clinical prognosis [3–6]. This is likely due to the fact that bone metastases are not usually detectable until advanced stages of disease when macrometastases have already formed, causing massive bone destruction. Moreover, bone can serve as a reservoir from which metastatic cells subsequently disseminate to other tissues, a process likely underway at diagnosis, further complicating clinical intervention [7].

When first posited in 1895, the “Seed and Soil hypothesis” suggested that cancers preferentially metastasize to specific secondary sites depending on their primary origin and that metastasis of a given disseminated tumor cell (DTC), “the seed,” is facilitated by the local conditions, “the soil,” of a secondary site. A seed will only grow if the soil is permissive [8]. It is now widely appreciated that primary tumor cells from the breast can spread to bone very early during disease development and enter a state of latency for years or decades (or sometimes forever) before recurring as metastatic tumors [7, 9, 10]. Indeed, non-proliferative DTCs have been detected in the bones of patients who were in remission from a primary cancer and succumbed to another ailment. These so-called dormant cells are often resistant to chemotherapy and radiation, which motivated the research that led to the identification of multiple molecular pathways and conditions that regulate cancer cell dormancy and chemotherapy resistance including altered expression or activity of signaling nodes NR2F1, ERK, p38/MAPK, and MSK1, as well as hypoxia [11–13]. However, tumor cell extrinsic factors are similarly important in both tumor cell dormancy and reactivation but much less well understood.

Efforts to understand how the microenvironment of a future metastatic site, often termed the premetastatic niche, dictates dissemination, dormancy, and outgrowth have identified different cellular and biochemical elements that play a role in these processes including tissue-specific host cells, immune cells, and cell-secreted morphogens such as soluble factors and extracellular vesicles (EVs) [14, 15]. Moreover, physical cues such as cell stiffness, tissue mechanical properties, and the dynamic coupling between both are important [16–19]. How changes of the extracellular matrix (ECM) impact these different signaling mechanisms and thus, the pathogenesis of bone metastasis remains largely unclear despite the well-known role of ECM in defining the skeleton’s hallmark biological and mechanical functions [20].

The large majority of bone (approximately 90–95% of bone’s dry weight) is composed of ECM, and decreased bone density is a risk factor for bone metastasis [21, 22]. Hence, it is likely that changes to bone matrix play a critical role in regulating early stages of bone metastasis. In support of this hypothesis, researchers have found that factors decreasing bone mineral density such as chemotherapy or menopause worsen the risk for breast cancer bone metastasis [23, 24], while increasing bone density (e.g., through exercise-based mechanical loading of the skeleton) protects against metastasis [25, 26]. When matrix changes, both host cells and DTCs are subject to dynamic levels of stiffness and elasticity, altering integrin-dependent mechanosignaling and ultimately cell phenotype. Although research of primary cancers or metastasis to soft tissue organs indicates that the ECM is a key regulator of progression [27, 28], little is known about how changes of bone ECM affect metastatic colonization or outgrowth in the skeleton. The limited knowledge is due in part to a lack of model systems that allow selective control over bone matrix’s biochemical and biophysical properties. In this review, we discuss the role of bone matrix in regulating cell signaling in the bone metastatic microenvironment. We highlight current engineered model systems that provide insights into cellular interactions with healthy and diseased bone matrix, and outline design considerations for natural and synthetic systems that enable precise manipulation of the bone microenvironment and investigations of mechanisms controlling bone metastasis and therapy resistance.

## The bone microenvironment

### Bone extracellular matrix

**Fig. 1:**
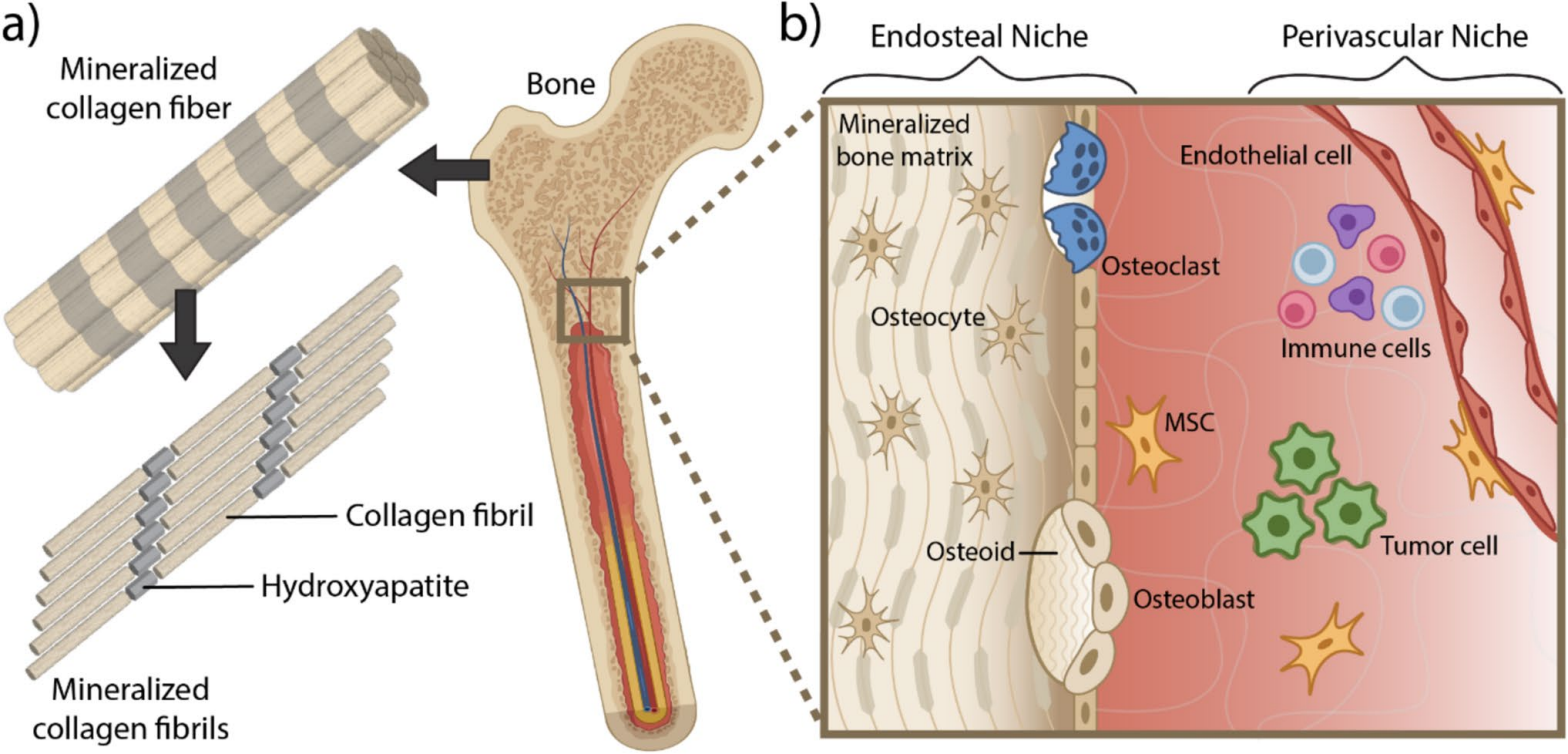
The ECM and cellular microenvironment of bone. **a** Bone is a hierarchical tissue whose unique biomechanical functions are defined by its basic building block, collagen type I fibrils embedded with carbonated hydroxyapatite (HA) nanoparticles. **b** Tumor cells disseminate from the primary tumor within the breast and colonize the bone, interacting with different niches and cell types. In the endosteal niche tumor cells interact with mineralized bone matrix and resident bone cells including osteoblasts, osteoclasts, osteocytes, and osteogenic precursors including mesenchymal stem cells (MSCs). The perivascular niche surrounds blood vessels, maintains hematopoiesis and production of blood and immune cells, and is also a source for MSCs

Bone is a complex and hierarchically structured tissue consisting of an outer layer of dense cortical bone that surrounds a mesh-like network of trabecular bone with interstitial cavities containing bone marrow. The basic building block of bone, collagen type I fibrils embedded with carbonated hydroxyapatite (HA) nanoparticles, endows the skeleton with its characteristic strength and toughness (Fig. 1a). During new bone formation, osteoblasts first deposit collagen type I-rich osteoid that is subsequently mineralized. During osteoid deposition, collagen type I triple helices self-assemble into parallel-staggered collagen fibrils in which individual collagen molecules are shifted by about 67 nm yielding the typical collagen banding pattern detectable by electron microscopy [29]. Collagen is a viscoelastic material, whose mechanical behavior is time-dependent and further affected by covalent crosslinking of collagen molecules during tissue maturation [30]. Moreover, the unique structure of collagen fibrils directs HA nanocrystal formation, organization, and distribution, which can independently affect bone matrix mechanical properties [31, 32]. The dominant type of mineralization in bone is intrafibrillar, with secondary interfibrillar mineralization, but various extracellular parameters can impact this sequence including osmotic equilibrium and electroneutrality [33] with effects on mineral particle size, crystallinity, and calcium-phosphate ratio and thus bone mechanics [34].

Importantly, bone matrix mineralization varies as a function of anatomical location, disease state, age, and diet [23, 35–40]. For example, regions in bone where overt metastases typically develop are characterized by increased remodeling, more disorganized and immature HA, and reduced HA content overall compared to other regions [41, 42]. These findings also correlate with the location of patient metastases that occur primarily in trabecular regions of bones that are highly vascularized and experience higher rates of bone turnover (e.g., spine, ribs, pelvis, and upper parts of long bones) [43]. Moreover, conditions in which bone matrix formation is perturbed (e.g., due to callus formation following fracture healing) increase metastatic colonization [44, 45]. As clinical evidence suggests that reduced bone mineral density increases the risk for developing breast cancer bone metastasis, a connection to bone matrix changes is evident.

### Effect of bone matrix mineralization on mechanotransduction

In the absence of mineral, cells interact with the collagen-rich bone matrix primarily via integrins, which mediate adhesion and trigger intracellular signaling cascades. Binding of β1-integrins including α1β1, α2β1, and α11β1 to distinct motifs within collagen fibers leads to integrin clustering and the formation of focal adhesions, which link collagen to the actin cytoskeleton and activate signaling pathways that regulate cell migration, proliferation, survival, and differentiation. These outside-in and inside-out signaling networks are dynamic and reciprocal as cells can remodel collagen through force generation and matrix metalloproteinase (MMP) activity [46–48]. The resulting changes not only affect cell phenotype via exposure of cryptic binding sites but also lead to tension-driven collagen fiber alignment and long-range force transmission [49]. The ensuing stiffness feedback influences integrin engagement and cell signaling and thus mechanical communication between cells over long distances.

**Fig. 2:**
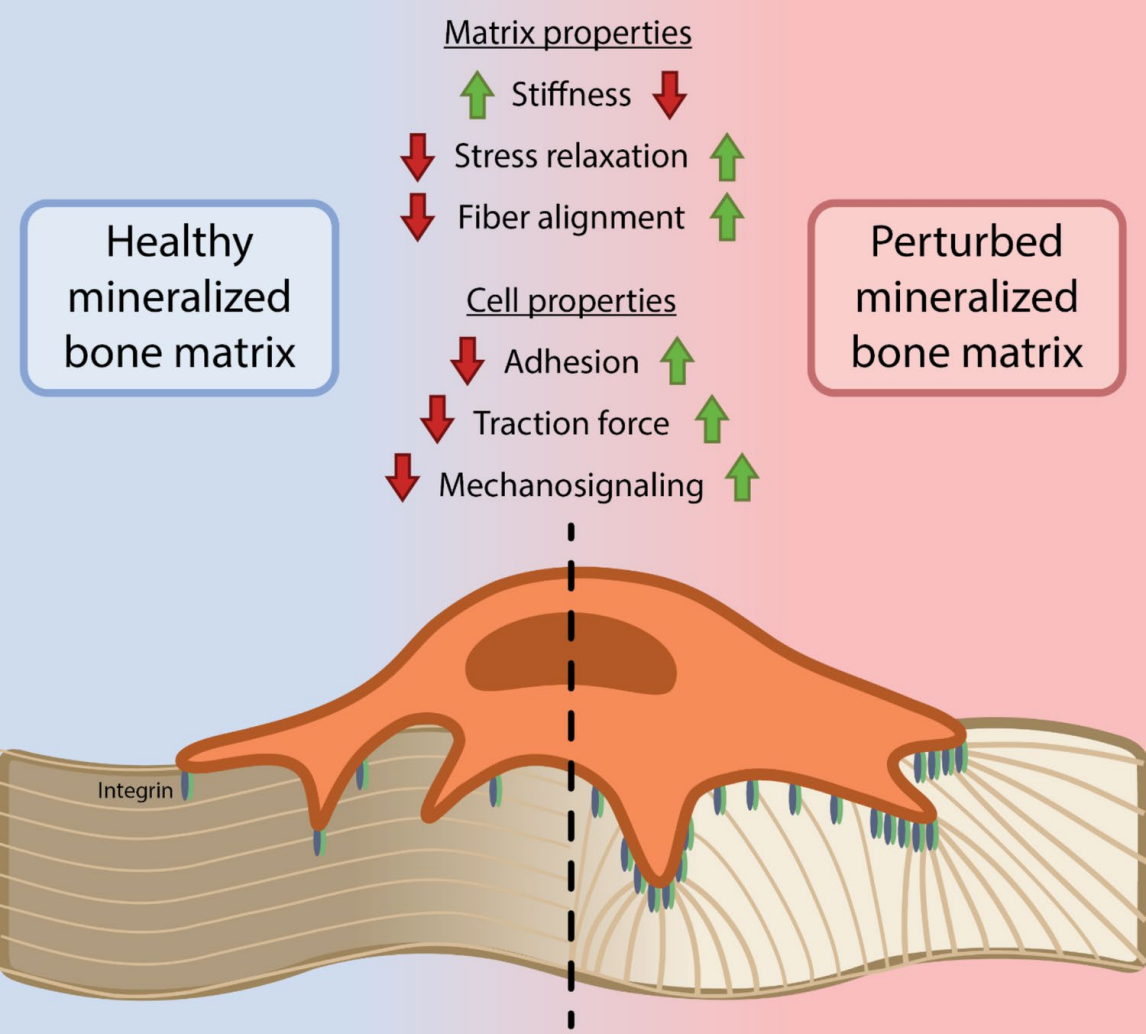
Bone matrix mineralization effects on mechanobiology. The mechanical properties of bone matrix change as the collagen fibrils are mineralized to form bone. Mineralization increases ECM stiffness while reducing stress relaxation which hinders the cell’s ability to align the matrix fibers. Cells on mineralized bone matrix experience decreased adhesion and traction forces that dampen integrin-mediated mechanosignaling

Mineralization of bone matrix influences these signaling mechanisms in various ways. For example, mineralization alters the viscoelastic properties of collagen, such as strain stiffening and stress relaxation, which can independently impact cancer progression [50, 51] and affect collagen fiber alignment, stiffness feedback, and ultimately integrin-mediated mechanosignaling (Fig. 2) [52, 53]. Indeed, breast cancer cells precultured on mineralized collagen exhibit reduced adhesion forces when quantified via traction force microscopy compared to cells precultured on collagen alone [53]. These observations are somewhat unexpected, given that mineralization increases matrix stiffness and that cells use the same β1-integrins to engage both mineralized and non-mineralized collagen [53]. However, they may be explained by mineral-mediated changes in matrix viscoelasticity and in the availability of collagen binding sites, which together influence integrin clustering, downstream signaling, and subsequent cellular traction forces. In addition, incorporation of HA into collagen affects the adsorption and conformation of serum proteins, such as fibronectin, with potential independent effects on cell signaling and tumor cell dormancy [54, 55]. Finally, it is possible that a local decrease in pH, that is, for example, mediated by the aerobic glycolytic metabolism of DTCs, leads to dissolution of mineral and thus an increase in extracellular calcium that can independently affect cell behavior [56, 57]. Better understanding how collagen mineralization regulates calcium signaling is important as this pathway can also be activated by the opening of calcium channels on the plasma membrane of cells experiencing increased tension [58]. Hence, calcium signaling could be enhanced on both collagen and mineralized collagen, but different mechanisms might be at play.

### Cell responses to bone matrix mineralization

As disseminated tumor cells localize to bone, they first enter the marrow compartment where they interact with two major stem cell niches regulating the bone microenvironment (Fig. 1b). The first, the perivascular niche, is proximal to blood vessels in the marrow. This niche is the primary site of hematopoiesis in the body including leukocytes such as monocyte-derived macrophages and osteoclast precursors [59]. The second niche is the endosteal niche [6, 60, 61] which maintains homeostasis of the bone matrix and is primarily composed of bone-forming osteoblasts, osteocytes, and distinct populations of skeletal stem cells [62, 63], often broadly referred to as mesenchymal stem cells (MSCs), as well as bone resorbing osteoclasts. Additionally, immune cells like natural killer cells and T cells periodically patrol both niches. Although perivascular and endosteal niches are often investigated separately, it is clear that cross-communication between both is involved in the pathogenesis of metastasis.

#### Effect of bone matrix on tumor interactions with bone resident cells

Because cell signaling within microenvironmental niches is inextricably linked to the matrix with which cells interact, altered bone mineral content naturally changes the cellular composition of endosteal niches with functional consequences on metastatic progression. For example, collagen mineralization in endosteal niches decreases breast cancer cell proliferation while promoting a quiescent, stem-like phenotype, possibly contributing to metastatic latency in patients successfully treated for primary breast cancer [53]. However, changes in the bone matrix affect not only tumor cells but also bone resident cells. For example, increased matrix stiffness and stress relaxation promote osteogenic differentiation of MSCs compared to softer materials with slow stress relaxation [64, 65], and MSCs cultured on mineralized substrates are more prone to osteogenic differentiation than their counterparts on non-mineralized conditions [66–68]. These matrix-dependent effects extend to interactions with tumor cells: tumor cells proliferate less when co-cultured with mineral-depositing, osteogenic MSCs versus MSCs that lack mineralization [53]. Interestingly, reduced osteogenic differentiation of MSCs is accompanied with increased fibronectin deposition, a factor that can independently regulate bone metastasis and dormancy [53, 55, 69]. A similar pattern is observed in fibroblasts, which, when exposed to non-mineralized ECM, acquire profibrotic and myofibroblast-like characteristics, phenotypes that are associated with worse prognosis in cancer patients [70, 71].

The interaction between cells and the bone matrix is bidirectional: as the matrix guides cell behavior, cells also modify the matrix. For example, tumor cells degrade bone matrix via chemical and enzymatic means, contributing to the vicious cycle between cancer cells and osteoclasts that increases tumor progression and bone resorption [57, 72–75]. Although MSCs and their progeny, osteoblasts, are typically defined as “bone-forming” cells, they can also encourage bone lesions [60, 62, 76–80]. Indeed, parathyroid hormone-stimulated RANKL secretion by osteogenic cells in the endosteal niche increases osteoclastogenesis and osteolysis [6, 81, 82]. In addition to these cell types, osteocytes also warrant attention. As the primary mechanosensors in bone, osteocytes play a central role in orchestrating bone matrix remodeling and maintenance. For instance, load-induced mechanical stimuli generate interstitial fluid flow within the bone activating osteocytes to promote the osteogenic differentiation of MSCs [83]. Accordingly, force generated from weight-bearing exercise increases bone formation and inhibits tumor growth [25, 26, 84–86], while the absence of mechanical stimuli results in osteocyte apoptosis ultimately leading to osteoclast activation as well as increased bone turnover and tumor cell proliferation [84–87]. The presence of cancer disrupts the interconnected osteocyte network embedded within the mineralized bone matrix [88] potentially impairing bone matrix mineralization by altering cellular responses to mechanical stimuli. Such disruptions in mineralization may, in turn, compromise the mechanical integrity of tumor-bearing bone [89] and ultimately lead to metastasis-associated fractures.

#### Effect of bone matrix on tumor-immune cell interactions

Because cancer cell dormancy and progression are at least in part mediated by their ability to escape immune attack [90], it is also important to consider how bone matrix properties regulate tumor-immune cell interactions. Interestingly, a recent comprehensive analysis suggests that the immune characteristics of bone metastases do not stem from their primary tumor but are more likely to result from interactions with the bone microenvironment [91]. This study also found that skeletal stromal cells (including MSCs, myofibroblast-like cells, and osteoblasts) are key drivers of bone metastasis progression [91]. Given the critical role of these cells in the remodeling of and response to bone matrix, it is likely that bone matrix is a key regulator of the bone metastasis immune environment.

Experimental evidence supports this point and suggests both direct and indirect mechanisms may be involved. Recent *in vitro* data indicate that tumor cells interacting with mineralized bone matrix are more efficient in evading immune surveillance [92]. For example, mineralized bone matrix alters their metabolism to increase the thickness of their glycocalyx, which, in turn, can function as a physical barrier against immune attack by natural killer (NK) cells [92, 93]. Interestingly, bone matrix mineralization may also regulate tumor-immune cell interactions through EVs as increased thickness of the tumor cell glycocalyx can drive EV biogenesis, a process that is further modulated by the stiffness of the local microenvironment [94, 95]. Therefore, it is likely that bone matrix mineralization controls interactions between tumor and immune cells via multiple different mechanism, but more studies are necessary to better understand their relationship.

In addition to these indirect effects, bone matrix can regulate hematopoietic stem cell (HSC) and immune cell phenotypes directly. For example, HSC maintenance and cell-fate decisions are determined by matrix stiffness and ligand composition [96], and mineral particle size, crystallinity, and topography regulate inflammatory responses by controlling macrophage and dendritic cell phenotype [97–99]. In the absence of mineralization, macrophages can also degrade collagen more readily, which alters their metabolism and polarization state to create a tumor-permissive environment [100]. Finally, lack of bone matrix mineralization contributes to a pro-tumorigenic environment via the activation and recruitment of tumor-associated neutrophils [70]

Taken together, the relationship between bone matrix, mechanotransduction, different cell types, and immunity plays a critical and yet poorly understood role in the initiation and progression of bone metastasis. Thus, model systems that capture the physicochemical properties, architecture, and cellular composition of bone matrix are essential to understanding its role in cancer biology and bone metastasis.

## Conventional models of bone metastasis

### *In vivo* models

As spontaneous metastasis to the skeleton is rare in commonly used mouse models of breast cancer (e.g., transgenic or xenograft models), bone metastases in mice are typically generated by injecting tumor cells directly into the tibia or into the arterial circulation [42]. Humanized mouse models of bone metastasis can be achieved by injecting human breast cancer cells into immunocompromised mice co-engrafted with human immune cells [101], whereas studies necessitating a fully intact immune system can utilize syngeneic immunocompetent mouse models with murine cancer cell lines and strain-matched mice. When combined with prior interventions to alter bone density, these models can yield new insights into how bone changes influence bone metastasis. For example, bone mineral density is reduced with aging [102], diet-induced vitamin D deficiency [39], chemotherapy treatment [23], or genetic models such as hypophosphatemic osteomalacia [103]. Vice versa, bone formation and thus mineral density can be increased by mechanical loading as described above [104]. While these approaches have increased understanding of the functional links between bone remodeling and metastasis, they also have systemic effects, including on the immune system, that obscure matrix-dependent specific effects [103, 105, 106].

The respective injection routes of tumor cells are subject to limitations as well. For example, intratibial injections cause local injury and healing responses that can influence tumor growth [107]. Moreover, they typically involve transplantation of high numbers of tumor cells that do not mimic clinical scenarios of bone metastasis in which small numbers of tumor cells seed and survive in the skeleton [6]. To overcome these limitations, mouse models of bone metastasis are commonly generated using intracardiac injection of breast cancer cells. While this injection route does effectively deliver tumor cells to trabecular bone, it also leads to systemic dissemination of tumor cells which often cause lethal metastases in off-target organs, such as the liver and lungs [108]. Alternatively, direct injection via the iliac or caudal artery forces tumor cells to bypass the lungs and delivers them directly to the hind limbs to reliably form metastases in the long bones of mice [109, 110]. Clinically, however, the majority of bone metastases occur in the axial skeleton (e.g., the spine and ribs) and therefore the injection methods described above only model some types of bone metastasis in humans [43]. Furthermore, these approaches are technically challenging, highly variable in results and require large sample sizes, and do not accurately model the earlier stages of the metastatic cascade. More generally, experiments combining these different methods are extremely lengthy and do not lend themselves for high-throughput approaches as needed for drug testing in a precision medicine setting.

### *In vitro* models

Efforts to develop *in vitro* models of bone metastasis have been extensive. Initial studies of bone metastasis at the metastatic site employed simplified 2D cell culture models on tissue culture plastic (TCP), but this approach does not adequately represent the complex architecture and material characteristics of bone tissue. Given bone’s unique matrix composition and its various effects on cell signaling as described above, it is suspected that these features regulate bone metastatic progression. More recently, *in vitro* models of bone metastasis have transitioned towards using 3D polymeric scaffolds and hydrogels to better mimic the 3D nature of the bone microenvironment [111]. However, these systems often lack the microscale fibrillar architecture and hierarchical organization that characterizes bone and defines its mechanical and biochemical properties. Furthermore, such models rarely consider the mineral component of bone matrix or do not account for HA properties that change depending on disease-, diet-, and age-related alterations of bone [106, 112–114]. Despite epidemiological and basic research findings connecting changes in bone density to metastatic progression, it is often overlooked in models of bone metastasis.

In addition, many *in vitro* models of bone metastasis utilize only tumor cells, overlooking the contributions of other bone resident cells in the pathogenesis of bone metastasis. For example, crosstalk between cancer cells and osteogenic cells can both promote tumor growth and osteolysis or induce tumor cell dormancy depending on microenvironmental cues. Additionally, vasculature and the immune system play a critical role in both the initial colonization of tumor cells in the bone and subsequent metastatic outgrowth. Thus, it is essential for models of bone metastasis to incorporate both physiologically relevant bone matrix and resident bone cells to advance our understanding of cell–cell and cell–matrix interactions that govern metastatic outgrowth.

## Engineered models of bone metastasis

### Hydroxyapatite nanoparticle models of mineralized bone matrix

As HA is the primary inorganic component of bone matrix that is essential for bone’s characteristic physical and mechanical properties, researchers have developed techniques to synthesize HA nanoparticles for incorporation into cell culture and tissue-engineered models. Originally established to improve bone regeneration, these approaches can recapitulate both the chemical composition and physical structure of HA nanoparticles to investigate their role in bone metastasis. For example, synthetic HA nanoparticles offer tunability of HA properties such as shape, size, and crystallinity and were first incorporated into 2D cell culture platforms to study how the physical properties of HA influence cell behavior [115]. Smaller HA particles and reduced HA crystallinity resulted in increased cell viability, proliferation, and adhesion of MSCs and osteoblasts, compared to cells cultured with larger and more crystalline HA particles [116–118]. Additionally, shape and carbonate content of HA particles have been shown to influence the metastatic potential of breast cancer cells [119]. Although this approach enabled the direct study of HA on cell behavior, it lacked the dimensionality and structure of bone matrix. It is well known that cells behave differently when cultured on 2D substrates than when they are cultured in 3D materials [120, 121], motivating the need to integrate HA with tunable 3D scaffolding to mimic the complex architecture of bone.

Successful attempts at addressing dimensionality questions in bone models have often leveraged polymeric scaffolds with embedded HA nanoparticles. Such scaffolds offer considerable tunability in terms of mechanical properties and porosity and can be made from a variety of natural and synthetic polymers. For example, the use of hydrogel scaffolds enables control over material stiffness and viscoelasticity by altering crosslinking type and concentration of the polymers [65, 122, 123]. Moreover, specific types of polymers can be selected based on the design parameters of the scaffold and overall goal of the model system. Synthetic materials are beneficial because of scalability, reproducibility, and customization while natural materials offer biocompatibility, remodelability, and biodegradability (Table 1). Combining these materials with HA nanoparticles, 3D scaffolds can be fabricated using processes such as electrospinning [124], 3D printing [137, 138], and solvent-casting particulate leaching [130, 131]. Breast cancer cells can then be cultured within these scaffolds to study HA interactions on cell behavior in a more physiologically relevant 3D environment. In particular, the effects of HA size and crystallinity on breast cancer cells were investigated using poly(lactide-co-glycolide) (PLG) scaffolds containing HA nanoparticles and it was found that small HA nanoparticles with poor crystallinity promoted adsorption of serum proteins and cell adhesion compared to larger and more crystalline HA nanoparticles [131]. While these models led to significant advances in understanding how HA influences cancer cell and stromal cell behavior, they ultimately lack collagen I, the core organic building block of bone, and physiological intrafibrillar mineralization that ensures bone’s unique biomechanical performance.

**Table 1:** Examples of natural and synthetic biomaterials for incorporation of mineral and mimicry of bone matrix biophysical properties

Material	Advantages	Disadvantages	Applications
Natural			
Alginate	Cost effective, high tunability, easily modified	Poor cell adhesion, poor mechanical strength	Electrospun fibrous scaffold [124], tunable-crosslinked hydrogel [65, 125]
Silk fibroin	High mechanical strength, intrinsic cell-binding sites	High rate of degradability	Electrospun fibrous scaffold [126], porous scaffold [127]
Chitosan	Good cell adhesion	Poor mechanical properties	3D-printed hydrogel [128]
Collagen	Natural ECM component, tunable architecture	High variability, poor mechanical properties	Hydrogel [52], cell-encapsulated hydrogel [129], porous scaffold [66]
Synthetic			
Poly(lactide-*co*-glycolide) (PLG)	High biocompatibility, biodegradable	Acidic byproducts from degradation cause HA dissolution	Porous scaffold [130, 131], mineralized film [132]
Poly(e-caprolactone) (PCL)	High mechanical strength, biocompatible, biodegradable	Poor cell adhesion	3D-printed scaffold [133, 134]
Polyethylene glycol (PEG)	High tunability, easily modified	Poor mechanical strength	Cell-encapsulated hydrogel [122, 135], 3D-printed hydrogel [136]

### Biomimetic mineralization of collagen hydrogels

**Fig. 3:**
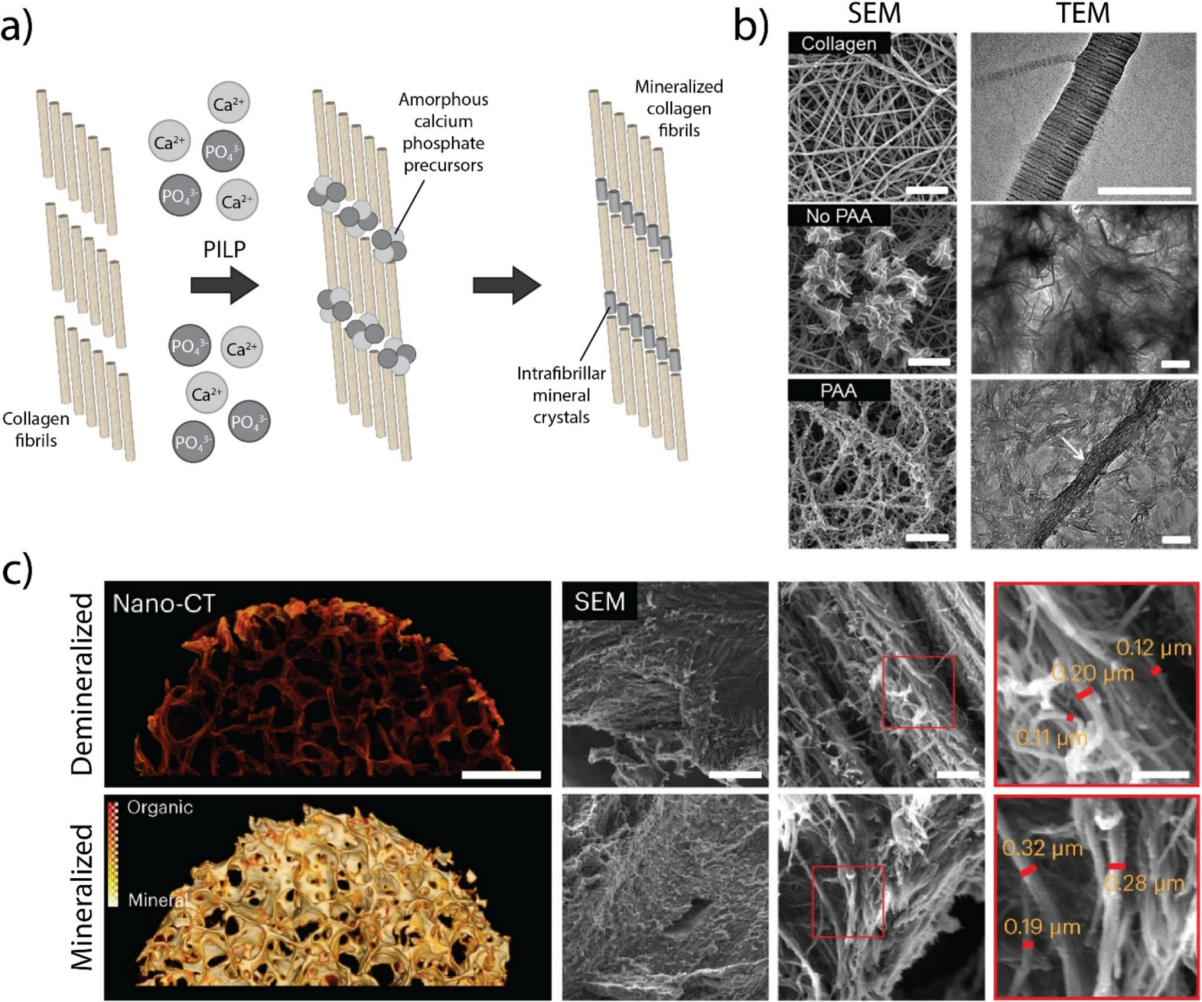
Biomimetic models of mineralized bone matrix. **a** The polymer-induced liquid precursor method (PILP) produces intrafibrillar collagen mineralization using a supersaturated solution of Ca^2+^ and PO_4_^3−^. Incubation with an acidic polymer, such as polyaspartic acid (PAA), enables penetration of amorphous calcium phosphate precursors into the collagen fibrils. **b** Transmission electron microscopy (TEM) of collagen fibers and scanning electron microscopy (SEM) of collagen after mineralization. Without the addition of PAA, only extrafibrillar mineralization occurs. Scale bar = 1 µm for SEM and 200 nm for TEM. Adapted from [52] with permission from Elsevier. **c** Nano-computed tomography (nano-CT) and SEM images of decellularized bone-derived scaffolds with and without prior demineralization. Bone matrix architecture and collagen fibrillar structure are retained after decellularization and demineralization. Scale bars = 1 mm for nano-CT and 10 µm (left), 2 µm (middle), and 1 µm (right) for SEM. Adapted from [53] with permission from Nature Portfolio

While fibrillar collagen type I, the main organic ECM component of bone, enables cell-mediated degradation and remodeling, many commonly used synthetic systems do not. Additionally, collagen hydrogels can be easily modified to alter scaffold density, porosity, and crosslinking and then mineralized, more faithfully simulating bone matrix properties. Attempts to mineralize collagen I began initially with the use of simulated body fluid (SBF), a blood plasma-like solution supersaturated with calcium and phosphate ions that allowed control over HA morphology and chemical composition [119, 132, 139]. This mineralization method facilitates the deposition of calcium phosphate minerals on the surface of collagen fibrils; however, it is not capable of achieving the intrafibrillar mineralization that occurs during secondary bone formation. This led to the development of the polymer-induced liquid precursor method (PILP) [31, 134] for mineralizing collagen hydrogels that recapitulate the intrafibrillar mineralization found in bone [31, 140]. The PILP method combines acidic polymers, such as polyaspartic acid, with a supersaturated solution of calcium and phosphate to create amorphous mineral precursors that penetrate collagen fibrils, producing intrafibrillar mineralization (Fig. 3a, b) [141]. Originally used for mechanistic studies of collagen mineralization, collagen hydrogels mineralized using the PILP technique have been adapted for cell seeding and routine culture to compare cell responses on collagen and HA-mineralized collagen [52, 142]. Culturing human breast cancer cells on collagen matrices with intrafibrillar mineralization was found to reduce breast cancer traction forces and proliferation [53]. However, cell culture studies using collagen mineralized via the PILP method only allow for 2.5D culture as the mineralization protocol does not permit simultaneous cell incorporation.

Aspiration to embed cells within a mineralized matrix to improve bone models has sparked the development of mineralization protocols that use osteopontin, an acidic non-collagenous protein found in native bone [143]. This approach has yielded cell-laden collagen hydrogels that can be mineralized in the presence of cells to create 3D cultures embedded with osteoblast-like cells and osteocytes for example [129]. Although these models recapitulate the composition of bone matrix and certain microscale characteristics, they lack bone’s hierarchical structure and associated bulk mechanical properties as well as other non-collagenous proteins normally sequestered in bone matrix. This is especially important as the structure and biochemical composition of organic matrix influences biomineralization, motivating the need for alternative scaffolds that replicate this complexity.

### Bone-derived scaffolds

In contrast to collagen hydrogel models, decellularized scaffolds harvested from mature bone can preserve the structural, chemical, and mechanical properties of native bone matrix. In addition to physicochemical properties, bone-derived scaffolds also include matrix-sequestered cell-secreted factors and can be seeded with cells of interest or complete marrow to simulate distinct aspects of the bone microenvironment [144]. Moreover, these scaffolds can be selectively demineralized to create a model with spatially controlled mineral content (Fig. 3c) [145–147].

Bone-derived scaffold models have been used to culture osteoblasts and osteoclasts to investigate osteogenic differentiation, bone remodeling, and osteolytic bone destruction. For example, mineral deposition by mature osteoblasts was mediated via organic matrix architecture and the degree of mineralization correlated with increased osteogenic differentiation of MSCs [148]. To study bone matrix mineralization effects on bone metastatic outgrowth, breast cancer cells were seeded on mineralized bone-derived scaffolds and exhibited a more stem-like phenotype and proliferated less compared to cells cultured on demineralized bone scaffolds [53]. A model of human bone marrow that incorporates both endosteal and perivascular niches was developed using osteoblast-laden bone scaffolds layered with a fibrin hydrogel containing MSCs, HSCs, and endothelial cells to investigate how microenvironmental cues regulate bone metastasis [149]. Interestingly, colonization of breast cancer cells resulted in a shift of hematopoiesis towards myeloid-lineage cell production, creating a more tumor permissive environment. Although bone-derived scaffolds recapitulate biochemical and physical properties of bone matrix, reproducibility concerns can arise due to the innate variability found in bone tissue depending on species and location from which the scaffold is sourced. Furthermore, some ECM components, such as glycosaminoglycans and growth factors, may be disrupted depending on the decellularization method used [150].

### Cell-derived matrices

Mineralized ECM produced by mature osteoblasts offers comparable biochemical and physical properties as native bone but makes imaging of cells easier relative to bone-derived scaffolds. Cell-derived matrices (CDMs) can be decellularized and then be re-seeded with different cell types to study how the CDMs affect their behavior [151]. Moreover, integrating CDMs with bioreactors and 3D scaffolds that degrade over time has resulted in multi-cell layered tissues to better replicate the physiological complexities of bone [152, 153]. The use of CDM systems yielded new insights connecting cellular crosstalk between osteoblasts, osteoclasts, and tumor cells to increased metastatic tumor growth that is further mediated by bone matrix resorption, confirming an important role of bone matrix in the vicious cycle and bone metastatic progression [154, 155]. These models include the main cellular and matrix components of bone, but do not offer precise control over other key factors within the bone microenvironment known to influence bone metastasis, such as vasculature, the immune system, and mechanical forces (flow, pressure, etc.).

### Microfabricated and bone-on-chip systems

By combining cell- and tissue-derived matrices with microfluidics and microfabrication techniques, researchers are engineering more sophisticated “bone-on-chip” microphysiological systems to investigate the bone microenvironment. These systems support 3D culture of multiple cell types and offer precise control over biochemical and biomechanical parameters, such as chemokine gradients and interstitial flow. Additionally, earlier steps of the metastatic cascade prior to the development of bone metastases can be modeled by incorporating vasculature to mimic the perivascular niche and investigate immune cell trafficking and tumor cell extravasation [156, 157]. For example, a vascularized immune model containing endosteal and perivascular niche components was developed to replicate the bone marrow and enabled the maintenance of HSCs and neutrophil trafficking throughout the vascular network [158]. Similar approaches have used tissue-derived scaffolds and controlled flow rates, shear stresses, and oxygen gradients to study bone colonization by cancer cells. Indeed, while interstitial flow-induced shear stresses on breast cancer cells inhibited cell proliferation, it also promoted drug resistance [159]. Recently, advancements in microfluidic technology have led to the development of a multi-organ chip system comprised of engineered heart, liver, bone, and skin tissue compartments connected via vascular flow to enable organ-organ crosstalk. Such systems are promising for future metastasis studies of cell–matrix interactions at the primary tumor site in breast tissue, dissemination throughout the vasculature, and colonization at the bone metastatic site [160].

### *In vivo* engineered implants and organoids

Although engineered *in vitro* models are powerful tools, it is not yet possible to capture the physiological complexity of a whole organism and the entirety of the metastatic cascade with these approaches. Implanting engineered model systems into mice may overcome this limitation and combine the physiology of a living organism with the customization and tunability of engineered systems to create dynamic experimental models with increased matricellular complexity. Multiple cell types can either be engrafted prior to implantation or recruited from the host after implantation into functionalized polymeric hydrogels or electrospun materials containing osteogenic factors to create bone organoids that model both mineralized bone matrix and the marrow compartment [74, 161, 162]. Bone organoids can also be achieved via direct transplantation of osteoprogenitor cells into the mouse kidney capsule, which effectively models the cartilage, stroma, and bone components of the microenvironment [163].

Using these approaches, cells derived from humans can be implanted into immunodeficient mice to create humanized models that more accurately reflect human bone physiology for translational research [164]. For example, human MSCs were engrafted onto collagen scaffolds and implanted into immunodeficient mice to create humanized miniature bone tissue that supported the homing and colonization of metastatic cells after systemic injection of human breast cancer cells [161]. Alternatively, syngeneic cells and immunocompetent mice can be utilized to investigate immune cell contributions towards metastatic outgrowth in bone [70]. Additionally, *in vivo* implantation systems enable intravital imaging of osteolytic lesion progression [74] and could provide an avenue to interrogate earlier stages of metastasis, such as the initial dissemination of tumor cells to a bone-like microenvironment. Collectively, tissue engineered implantation models can mimic some aspects of the complex bone microenvironment to study how interactions between cells and mineralized bone matrix may contribute to bone metastasis.

## Clinical relevance of engineered models of bone metastasis

While engineered models of bone metastasis provide valuable tools to gain new insights into disease mechanisms, integration of these platforms with precision medicine-inspired technologies will be necessary for analysis of patient-specific responses and widespread adoption of such platforms in clinical settings. For example, combining engineered bone matrix models with patient-derived organoids or xenografts will allow us to capture how bone matrix mineralization affects each patient’s unique tumor biology and tumor heterogeneity. Designing such models in multi-well formats to enable high-throughput screening using robotic liquid control systems would also drive the development of new and personalized therapies. For example, a microphysiological system could be developed to combine engineered bone scaffolds with various levels of mineralization and patient-derived organoids for which genomic and transcriptomic profiles have been collected previously. This approach will allow us to determine how bone matrix properties influence the progression of bone metastasis and which regulatory pathways may be involved while identifying potential drugs or combination of drugs that may be used to interfere with this process. The importance of such tools is likely to compound as the US Food and Drug Association (FDA) and other regulatory agencies increasingly emphasize the use of *in vitro* preclinical models for drug development and efficacy testing.

As early stages of bone metastasis are not clinically detectable, engineered models could also be valuable for the discovery of early biomarkers that predict the risk for breast cancer patients to develop bone metastasis later in life. Although current patient datasets are primarily collected from primary tumors, genomic and transcriptomic information from these specimens can be compared to those obtained from studies in bone metastasis models to evaluate patient outcomes. Indeed, gene expression profiles from breast cancer cells cultured on engineered mineralized versus non-mineralized bone matrix models mapped to gene signatures of patients that had better clinical prognosis, highlighting the critical role of healthy bone matrix and mineral density [53].

## Outlook

**Fig. 4:**
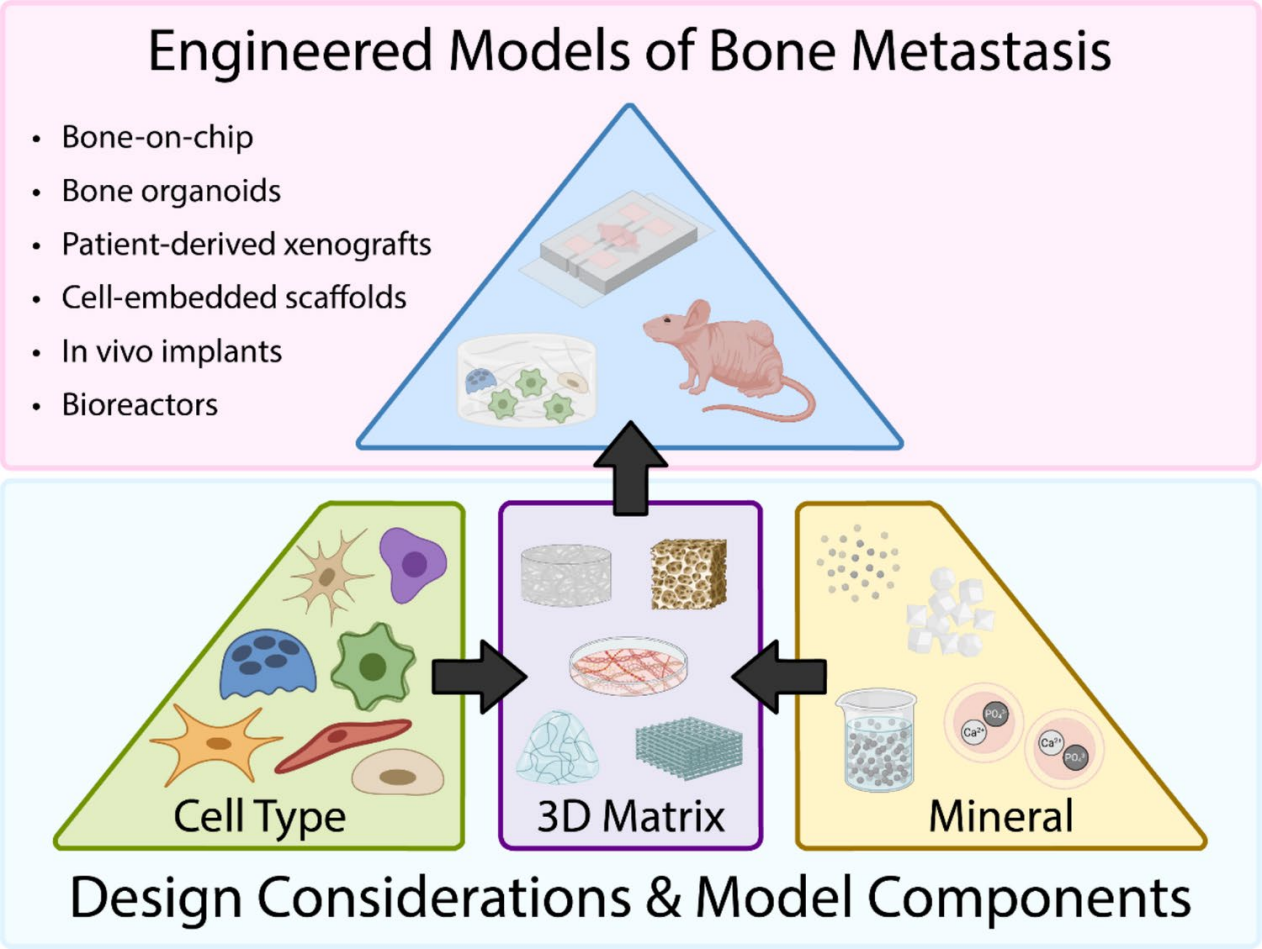
Design considerations for engineered models of bone metastasis. The three main components to consider when designing engineered bone metastasis models include cell type, 3D matrix, and mineral. Cell properties such as type, species, primary vs cell line, and co-culture abilities are key factors when selecting cells. Matrix mineralization can be achieved by cell-secreted vesicles containing Ca^2+^ and PO_4_^3−^ or in a synthetic manner using HA nanoparticles, simulated body fluid, and amorphous calcium phosphate precursors (e.g., polymer-induced liquid precursor method). Lastly, the 3D matrix can either be produced by cells themselves, such as cell- and tissue-derived scaffolds, or fabricated from natural and synthetic polymers to create cell-embedded hydrogels and constructs. Using these three design parameters, 3D models of mineralized bone matrix can be developed to study matrix-mediated effects on bone metastasis progression

Breast cancer bone metastasis is a complex clinical problem and current methods to treat and prevent bone metastasis are often ineffective long-term, significantly impairing the prognosis of patients with advanced disease. Although evidence suggests the composition of the bone matrix itself may be a prognostic factor, limitations of current *in vivo* model systems have slowed research in this area. Engineered model systems offer new tools to study the role of matrix in bone metastasis via selective control over inorganic and organic matrix composition, structure, mechanical characteristics, and cell types (Fig. 4). However, bone is a highly complex organ whose properties are not fully recapitulated by existing model systems. Further development of these systems to enable the inclusion of multiple cell types, precise control of mineral content, and incorporation of immune elements is warranted in pursuit of new clinical interventions. Additionally, the role of estrogen should be considered when designing future models as bone metastasis is mostly associated with breast cancer subtypes that express estrogen receptors and estrogen is a key regulator of bone remodeling [165].

Elucidating the specific regulatory role of bone matrix in metastatic cancer cell behavior using *in vivo* mouse models of metastasis or bone organoids is a unique challenge as the cell types implicated in bone metastatic progression also independently modify bone matrix properties. Additionally, MSCs and their progeny, osteoclasts, and other immune cells like macrophages, mechanosensing osteocytes, fibroblasts, and breast cancer cells all affect each other reciprocally by secreting soluble factors and EVs that affect cell–matrix and cell–cell interactions. The phenotype of many of these cells is highly plastic and itself is adapted to matrix physicochemical properties. For example, increased secretion of parathyroid hormone, stimulated in osteoblasts by interactions with tumor cells, promotes differentiation of osteoclasts that degrade bone matrix creating a mechanically softer microenvironment that is rich in soluble factors to promote additional tumor growth [82, 166]. Moreover, bone metastasis is a multi-step process that starts at the primary tumor within the breast and involves numerous organs and systems, each with their own extracellular matrix and cell types. Such complexities of the metastatic cascade cannot currently be modeled and require a new generation of microphysiological bone models that capture the inter-organ- or organism-level microenvironmental cues at play.

By leveraging technological advancements in additive manufacturing and soft-lithography, microfabrication, chemical and cell-mediated biomineralization, bioreactor design, and organ- and body-on-chip technologies, better mechanistic understanding of bone metastasis and treatment is attainable. Such technologies are rapidly being realized in other diseases and tissue contexts [167, 168] and would enable models that can capture the hormonal, hematopoietic, vascular, immune, osteogenic, adipose, and oncologic compartments of the metastatic tumor microenvironment while being analyzable via biochemical, molecular, or imaging means. For example, a humanized engineered model of mineralized metastatic tissue was established for the study of prostate cancer metastasis to bone [169, 170]. This advanced engineered *in vitro* system combines patient-derived xenografts with 3D-printed PCL scaffolds to mimic bone metastases with enhanced cellular and matrix complexity for investigating targeted therapies and drug responses. While initially developed for prostate cancer, these same techniques could be adapted for breast cancer studies to drive future innovation of engineered models for investigating bone metastasis. The development of new tools to model the metastatic bone microenvironment will enhance the understanding of how to prevent metastatic colonization and outgrowth and treat metastatic disease effectively in this highly complex and interconnected tissue.

## Data Availability

No datasets were generated or analysed during the current study.
